# Personalized medicine in a community health system: the NorthShore experience

**DOI:** 10.3389/fgene.2023.1308738

**Published:** 2023-11-28

**Authors:** Sean P. David, Henry M. Dunnenberger, Sarah Choi, Allison DePersia, Nadim Ilbawi, Christopher Ward, Dyson T. Wake, Janardan D. Khandekar, Yvette Shannon, Kristen Hughes, Nicholas Miller, Kathy A. Mangold, Linda M. Sabatini, Donald L. Helseth, Jianfeng Xu, Alan Sanders, Karen L. Kaul, Peter J. Hulick

**Affiliations:** ^1^ Department of Family Medicine, NorthShore University HealthSystem, Evanston, IL, United States; ^2^ Department of Family Medicine, University of Chicago Pritzker School of Medicine, Chicago, IL, United States; ^3^ Mark R. Neaman Center for Personalized Medicine, NorthShore University HealthSystem, Evanston, IL, United States; ^4^ Outcomes Research Network, NorthShore University HealthSystem, Evanston, IL, United States; ^5^ Department of Medicine, NorthShore University HealthSystem, Evanston, IL, United States; ^6^ Department of Medicine, University of Chicago Pritzker School of Medicine, Chicago, IL, United States; ^7^ Kellogg Cancer Center, NorthShore University HealthSystem, Evanston, IL, United States; ^8^ Department of Pathology, University of Chicago Pritzker School of Medicine, Chicago, IL, United States; ^9^ Center for Psychiatric Genetics, Department of Psychiatry and Behavioral Sciences, NorthShore University HealthSystem, Evanston, IL, United States; ^10^ Departments of Psychiatry and Behavioral Neuroscience, University of Chicago Pritzker School of Medicine, Chicago, IL, United States; ^11^ Department of Pathology, NorthShore University HealthSystem, Evanston, IL, United States

**Keywords:** personalized medicine, precision medicine, precision health, genetic counseling, genetic testing, pharmacogenomics, primary care

## Abstract

Genomic and personalized medicine implementation efforts have largely centered on specialty care in tertiary health systems. There are few examples of fully integrated care systems that span the healthcare continuum. In 2014, NorthShore University HealthSystem launched the Center for Personalized Medicine to catalyze the delivery of personalized medicine. Successful implementation required the development of a scalable family history collection tool, the Genetic and Wellness Assessment (GWA) and Breast Health Assessment (BHA) tools; integrated pharmacogenomics programming; educational programming; electronic medical record integration; and robust clinical decision support tools. To date, more than 225,000 patients have been screened for increased hereditary conditions, such as cancer risk, through these tools in primary care. More than 35,000 patients completed clinical genetic testing following GWA or BHA completion. An innovative program trained more than 100 primary care providers in genomic medicine, activated with clinical decision support and access to patient genetic counseling services and digital healthcare tools. The development of a novel bioinformatics platform (FLYPE) enabled the incorporation of genomics data into electronic medical records. To date, over 4,000 patients have been identified to have a pathogenic or likely pathogenic variant in a gene with medical management implications. Over 33,000 patients have clinical pharmacogenomics data incorporated into the electronic health record supported by clinical decision support tools. This manuscript describes the evolution, strategy, and successful multispecialty partnerships aligned with health system leadership that enabled the implementation of a comprehensive personalized medicine program with measurable patient outcomes through a genomics-enabled learning health system model that utilizes implementation science frameworks.

## Introduction

Since the completion of the Human Genome Project in 2003, the National Human Genome Research Institute (NHGRI) and other thought leaders set a bold vision to apply the advancement of genomic knowledge to address grand challenges in public health. The Grand Challenge II-5 investigated how genomic risk information is conveyed in clinical settings, how that information influences health strategies and behaviors, and how these affect health outcomes and costs (Collins et al., 2003). Twenty years later, the NHGRI’s strategic vision is to advance ‘virtuous cycles in human genomics research and clinical care’ between innovative genomics research, genomic learning healthcare systems, and new knowledge generation “to improve health at the forefront of genomics” (Green et al., 2020).

NorthShore University HealthSystem is an integrated healthcare delivery system including nine hospitals and a multispecialty group practice with over 140 Chicagoland locations. NorthShore accepted the grand challenge of advancing genomics into health (Green et al., 2011), starting with a feasibility study involving primary care and specialty physicians about their interest in and preparedness for offering genomic services (Selkirk et al., 2013). Results indicated perceived low levels of confidence to provide genomic services, while patient demand for these services was increasing.

NorthShore launched a decade-long investment to build an innovative, integrated, personalized medicine program grounded within a rapidly learning healthcare system model. The Center for Personalized Medicine (PMED), launched in 2014, has a strategic focus on primary care clinical implementation and research. We describe our efforts as focused on germline genomics, addressing risk assessment, stratification, and scaling personalized care in a seamless, sustainable manner across a community health system. The goal is to deliver on the promise of personalized medicine by utilizing genomics to preempt illness and precisely treat disease to improve the health of our patients and families.

## Methods

### Early vision and foundation for personalized medicine

**FIGURE 1:**
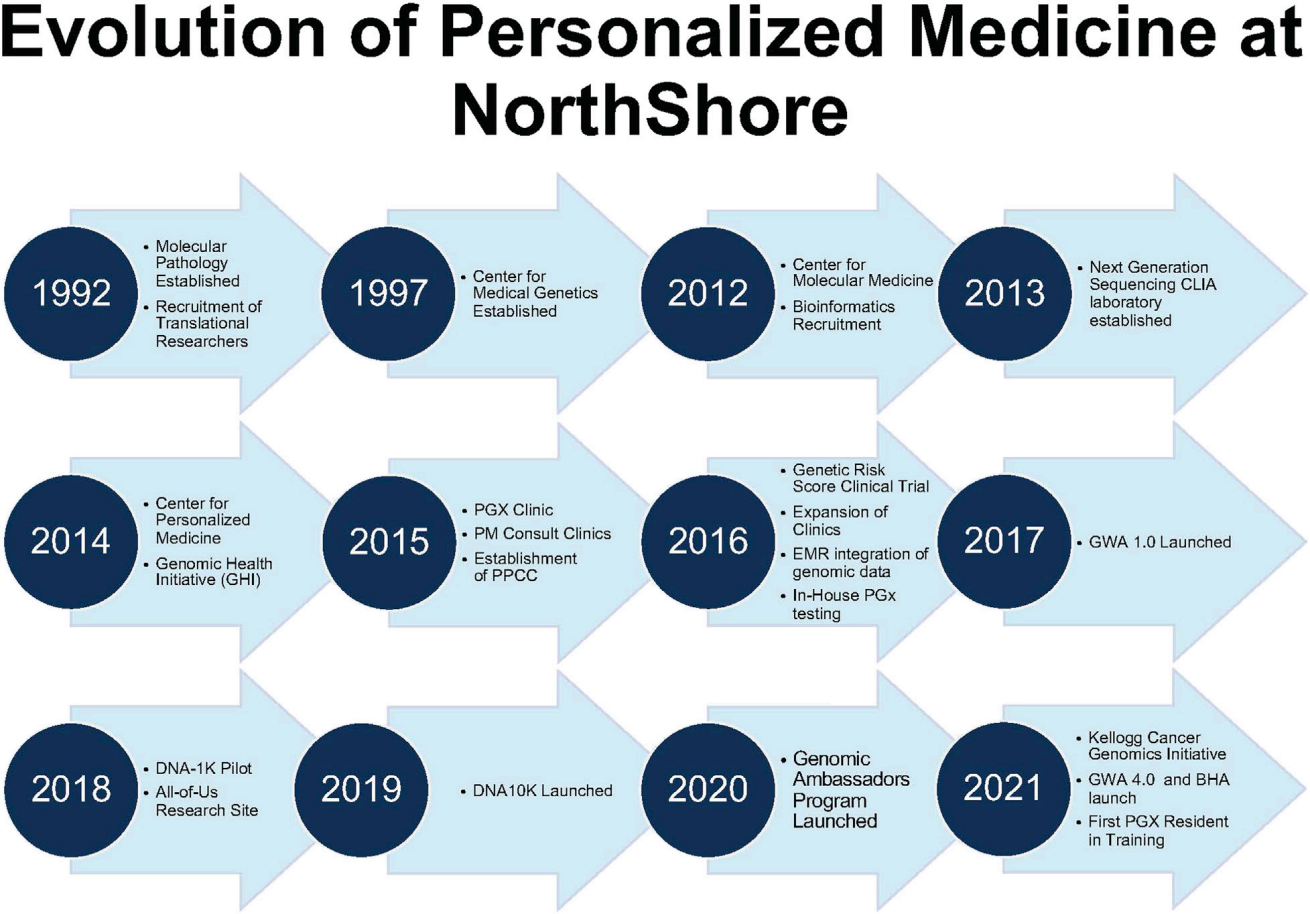
Evolution of personalized medicine at NorthShore. Timeline of Center for Personalized Medicine Developmental Milestones. BHA, Breast Health Assessment; CLIA, Clinical Laboratory Improvement Amendments of 1988; and GWA, Genomic and Wellness Assessment.

PMED’s vision is to solve the “last mile” of implementation. The foundation began in 1989 with a “Molecular Biology and Genetics” task force to address the challenges for a future of genomics-guided care. The “Molecular Medicine” model had four core elements: clinical genetics, screening, diagnostics (molecular/cytogenetics), and research. Molecular Pathology (1992) and the Division of Genetics (1997) created a critical foundation. Strong partnerships between clinical and administrative champions were key for continued success through this journey (Figure 1).

### Personalized medicine clinical and research infrastructure development

NorthShore invested in an institutional research biobank, the Genomic Health Initiative (GHI), that conducts translational, discovery research driven by the intersection of the electronic health record (EHR) and genomics data. Over 50,000 subjects have enrolled, and 30,000 DNA samples have been collected since 2014. The GHI’s key operational learnings include using the EHR patient portal to streamline participant outreach, education, consenting, and automating blood collection at the next phlebotomy appointment without impeding primary care. The high and signaled community interest in the GHI indicates a growing interest in genomics and health. The GHI’s participant outreach has enabled NorthShore to be a major partner in the Illinois Precision Medicine Consortium, which recruits participants for the National Institutes of Health (NIH)’s All of Us Research Program.

The development and clinical implementation of polygenic risk score (PRS) is a core translational research focus of PMED. Initial efforts focused on prostate cancer PRS and its clinical validity in multiple ancestral populations (Shi et al., 2021; Shi et al., 2022) and then expanded to additional conditions (Ahmed et al., 2022; Wei et al., 2022a; Glaser et al., 2022; Billings et al., 2023; Patel et al., 2023; Shi et al., 2023), including other cancer types (Shi et al., 2019; Northcutt et al., 2021; Wei et al., 2022b). Recently, our efforts have centered on the clinical implementation of PRS.

The Program for Personalized Cancer Care (PPCC) was implemented to improve the quality of cancer care through proactive, personalized care, from cancer prevention and screening to customized treatment of localized and advanced cancer with a focus on prostate cancer care. The Kellogg Cancer Genomic Initiative (KCGI) is a multidisciplinary program focused on the implementation and incorporation of NGS technology to better molecularly characterize cancer. Both are translational programs bridging the bench-to-bedside gap.

A pharmacogenomics (PGx) clinic (2015) was established with a multidisciplinary team to oversee the quality of testing, clinical guidance, and implementation, as described elsewhere (Dunnenberger et al., 2016). Scaling pharmacogenomics into primary care was an early priority given clinicians could relate to the concept that medications might work differently based on one’s genetics and privacy concerns were minimal. PCPs were offered testing through a pilot program to gain experience before broader implementation. Patient and physician experiences were evaluated, which provided critical learnings for scaling genomics in primary care (Lemke et al., 2017; Lemke et al., 2018).

### Development of FLYPE and EPIC integration

FLYPE is an in-house web-based bioinformatics platform that addresses challenges with the integration of personalized medicine (Helseth et al., 2021). FLYPE maintains NorthShore’s clinical knowledge base and updates variant annotations as new scientific knowledge emerges. Further integration into patients’ EPIC (Verona, WI) EHRs and their clinical decision support (CDS) capabilities has been implemented system-wide. The genomic indicator (GI) functionality of EPIC is one mechanism for capturing clinically significant variant data (e.g., pathogenic germline variants and pharmacogenetic variants) and their clinical implications and for triggering CDS alerts. The educational CDS messaging was previously only available to healthcare providers, but it has recently been “turned on” for patients as well to improve their knowledge and engagement with genomic data.

Over 702,000 GIs have been assigned to over 38,000 patients for CDS. Pharmacogenomics represents the largest GI dataset (over 695,000 indicators) assigned. As of 1 August 2023, over 390 CDS PGx rules are utilized, powered by 165 different PGx GIs covering 50 genes supporting the Food and Drug Administration, Clinical Pharmacogenomics Implementation Consortium (CPIC), and the Dutch Pharmacogenetics Working Group guidance. CDS embedded in the EHR has been a focal point for education and informing patients and clinicians about clinical advances in the field (Pritchard et al., 2021).

### Genetic and Wellness Assessment

PMED engaged widely with physician stakeholders for the implementation of direct access to PGx testing through primary care (Lemke et al., 2017). This crucial engagement laid a foundation for an EHR-integrated family health history tool, the Genetic and Wellness Assessment (GWA). The GWA began with a community educational event that invited participants, whether a NorthShore patient or not, to learn about personalized medicine. A simple “yes/no” questionnaire covering key core National Comprehensive Cancer Network^®^ (NCCN) indications for hereditary cancer risk was provided. Many elected to pursue follow-up, which signaled the need to improve access and awareness around hereditary risk assessment in our community.

**FIGURE 2:**
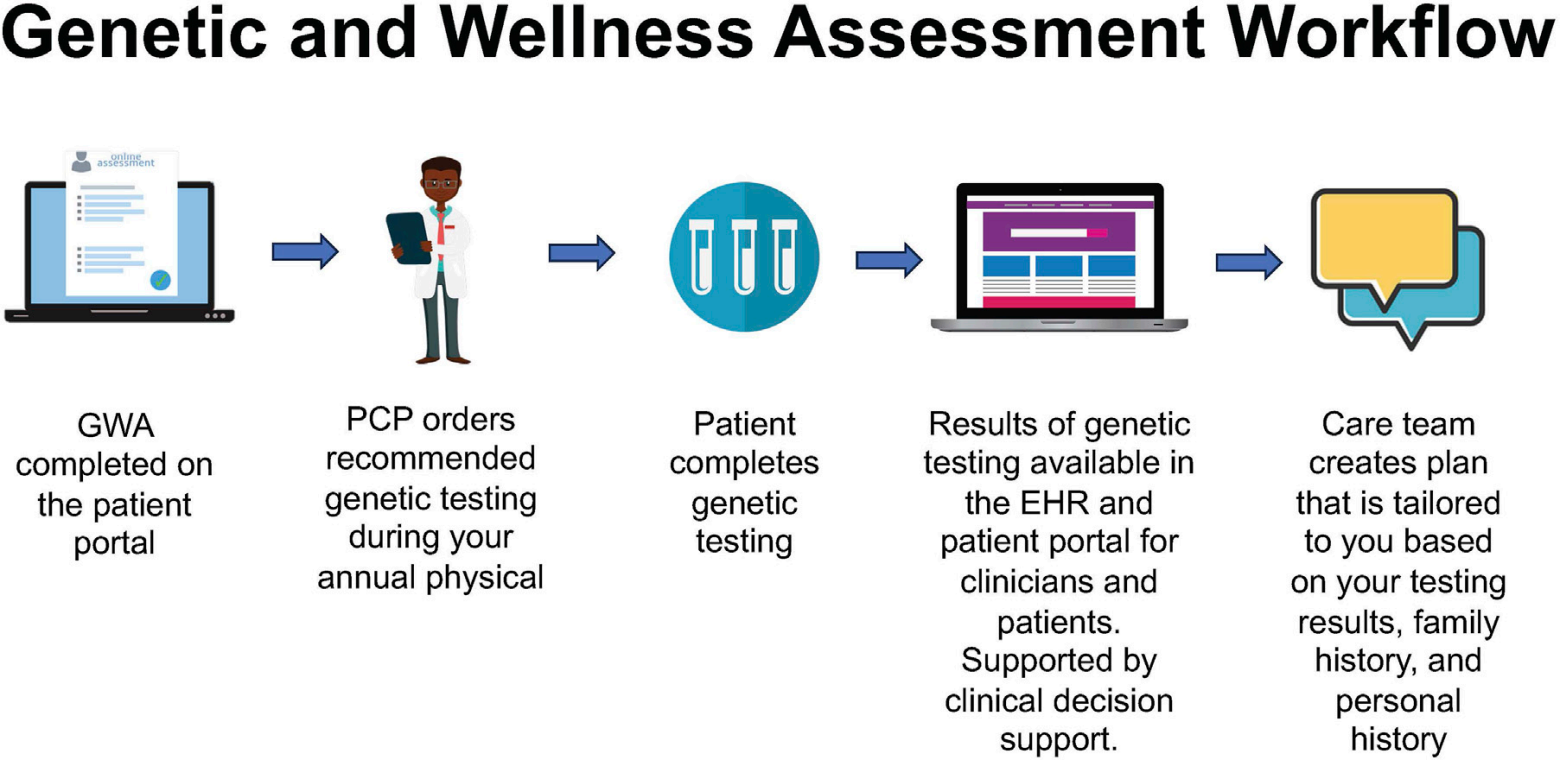
Genomic and Wellness Assessment workflow, starting with patient-facing registration and history, proceeding to primary care provider test ordering and clinical decision support, resulting in the return of results and development of a tailored care plan.

**FIGURE 3:**
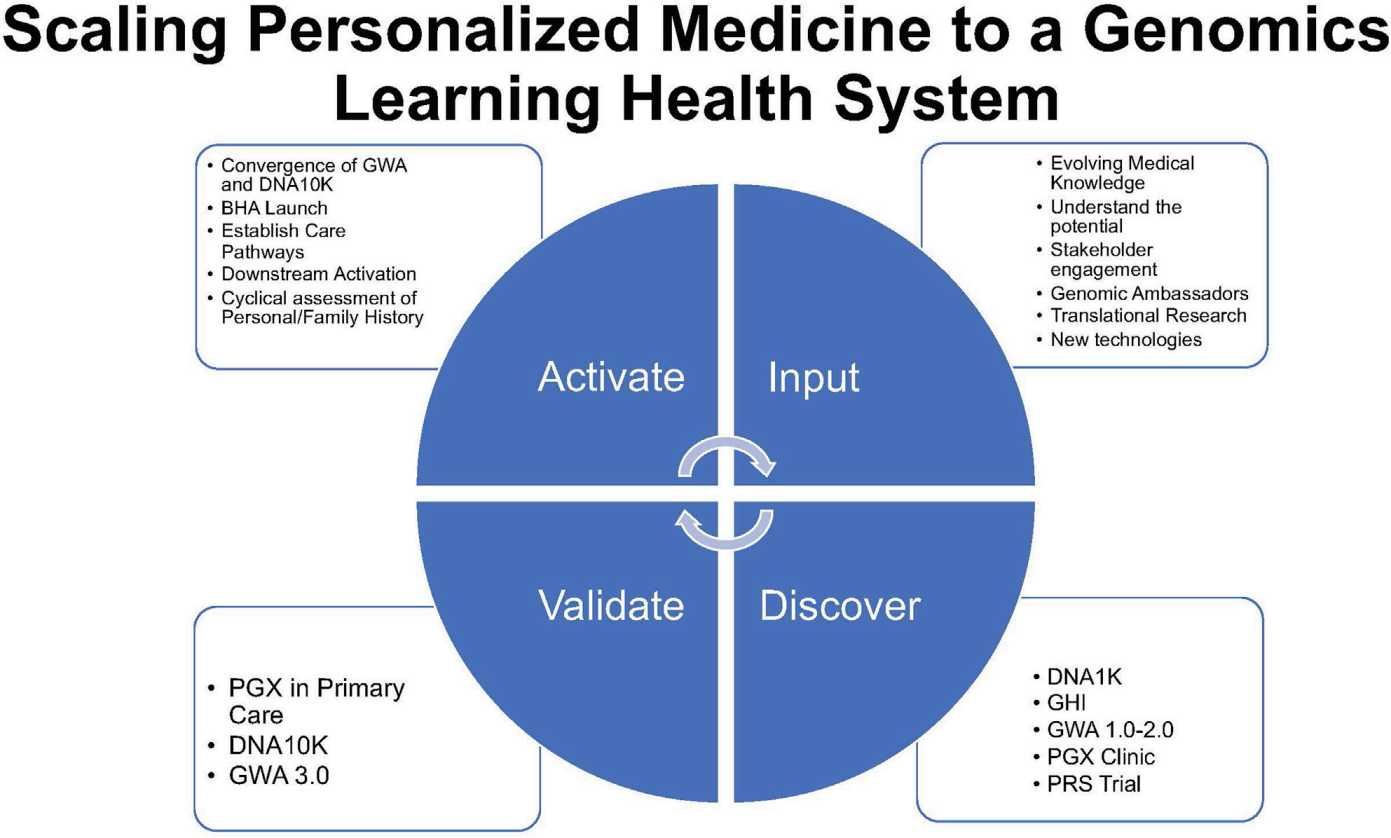
Scaling personalized medicine to a genomics learning health system represents a virtuous circle of activation, input of innovation, validation, adoption, adaptation, and renewal (activate–input–discover–validate) of the NorthShore implementation model.

The GWA assesses personal/family history at one’s annual history and physical appointment (Figure 2). Through the electronic check-in appointment process, patients complete the GWA along with routine pre-visit items (e.g., verifying health insurance, medications, and depression screening). Having the GWA as part of the routine, standard workflow was a goal. The GWA provides evidence-based guidance for genetic testing by identifying patients at higher risk for inherited conditions. The GWA has undergone a series of iterations guided by stakeholder feedback following the model of a learning healthcare system (Wouters et al., 2020) (Figure 3).

GWA 1.0 (2017) was the broadest iteration with 30 “yes”/“no” screening questions, with branching logic related to inherited cancer risk, cardiology, neurology, and endocrinology. Answers were prepopulated from EHR data. CDS alerts provided educational information in the line of care, prompting PCPs to consider genetic testing (e.g., hereditary cancer panel) or specialty referral or consideration of a “healthy gene panel” if targeted testing was not indicated. Specific specialists have been identified as genomics champions and content experts (Hulick et al., 2018). GWA 1.0 relied on physicians to act on Best Practice Alerts (BPAs), which had concise information regarding why an alert was fired and the rationale for the next step to be considered (e.g., referral and genetic testing). An internal review of 2018 data noted a 22% action rate on over 50,000 BPAs. Stakeholder feedback identified the following challenges: time necessary to complete the GWA, workflow as patients could fill out a paper alternative, and patient understanding of questions. Benefits included improved access to family history assessment and genetics. Based on the insights generated, iterative changes were made to the GWA (Lemke et al., 2020a). These included operational improvements with an emphasis on pre-visit workflows, reduction of the total number of questions, and limiting the domains to cancer and cardiovascular conditions. The GHI electronic consent and automated blood draw order signed by the principal investigator were implemented. BPA information was expanded based on the National Comprehensive Cancer Network^®^ (NCCN) guidelines. Following the successful pilot of the two medical group sites, full medical group deployment of GWA 2.0 was carried out.

### Population offering of genetic and pharmacogenomic screening (DNA1K and DNA10K)

While primary care physicians valued the GWA’s clinical utility, they recognized the limitations of an approach based solely on family history (Lemke et al., 2020b). The GWA (1.0–2.0) remained labor intensive, given a requirement to “act on” a BPA. A more patient-driven, “easy button” approach was piloted to remove traditional genetic testing barriers: cost, gating testing based on family/personal history, and time constraints of a primary care clinic (Supplementary Figure S1). Key to an “easy button” approach was stakeholder agreement that informed consent be captured electronically prior to the appointment.

To assess interest, system feasibility, and patient outcomes, as part of a learning healthcare system initiative, complimentary hereditary cancer genetic testing (30-gene next-generation sequencing-based panel) was offered through four primary care sites during any clinic visit, ranging from an acute illness visit to a patient’s annual exam. A total of 1,006 patients underwent testing in November and December of 2018 through DNA1K. An EHR review of these patients found that 92 pathogenic variants were identified in 90 (9.1%) patients in 16 genes (*MUTYH, CHEK2, APC, BRCA2, BRCA1, ATM, BARD1, MITF, BRIP1, NBN, PALB2, PMS2, RAD51C, MSH6, CDH1,* and *CDKN2A*). The high yield of positive tests and uptake of cancer screening test recommendations demonstrated the potential clinical utility of population-based screening programs. More patients completed genetic testing in 2 months than in the 18 months prior to the GWA (Supplementary Figure S2). DNA1K resulted in the launch of a coordinated care center to facilitate efficient patient follow-up through medical referrals and recommendations and the creation of a PCP advisory group (“Genomic Ambassadors”), and mechanisms were created to provide continual quality improvement and operational feedback to providers (Lemke et al., 2019).

NorthShore scaled DNA1K to DNA10K at 13 NorthShore Medical Group primary care sites spanning the health system’s geography. This was a pragmatic shift to focus on “proactive” screening rather than relying on “reactive genetic testing” based on personal or family history. Personalized medicine strategies were systematically implemented within primary care through DNA10K, thereby streamlining processes and physician practices (David et al., 2021a). Over 100 PCPs ordered Color Extended test panels for more than 10,000 patients who participated from early 2019 through 2020. Patients had the option to gain access to “fun traits” related to genomics, including ancestry, through Color’s patient portal to better understand the influence of direct-to-consumer offerings in the genomics space. Patients’ experiences with DNA10K were highly positive (Lemke et al., 2021). A subset of medical group primary care sites not part of DNA10K piloted further refinements of GWA 2.0, leading to GWA 3.0, which included “A/B” testing of customer relationship management (CRM) messaging prior to a patient’s visit, narrowing the line of questions to hereditary cancer, and development of a “more information” option for the patient to select after completing the GWA. This approach provided a method to schedule a complimentary genetic counseling assistant, a new position created based on feedback from primary care, visit under the supervision of the PMED team. This helped provide targeted discussion and triage of patients while relieving some of the burden on primary care physicians.

As a result of the iterative learnings from GWA 2.0 and DNA10K, the programs were merged to create a uniform primary care experience in January 2020, GWA 3.0 (Pritchard et al., 2021). Modeling the GHI consenting process and experience, patients were able to consent for testing with an order automatically placed in the PCP order set for the patient encounter. If the PCP did not override the order, it was automatically signed at the close of the EHR patient encounter and then released for sample collection and testing. To improve sustainability and clinical coverage, in GWA 4.0, patients who had a personalized or family history of cancer that likely met insurance coverage requirements were offered a hereditary cancer panel billed to insurance by our laboratory partner. Other patients were offered a population genetic screen with no insurance billing.

The GWA tool has undergone several refinements and has become more focused on hereditary cancer risk. The tool’s clinical validity is the highest when accessing this risk, and the gene content has been adjusted accordingly. The Breast Health Assessment (BHA), which is an adaptation of the GWA, focuses on the hereditary risk of breast and ovarian cancer types and is offered with screening mammography to capture a larger fraction of the population potentially at risk for cancer.

The current GWA version 4.0 (and analogous BHA) has been completed by over 150,000 individual patients, over 30,000 patients have screened “positive” on the personal/family history collection, and over 7,800 have undergone genetic testing through this primary care initiative. Similar success has been achieved with the BHA, with over 33,000 patients completing, 8,000 screening positive, and 2,300 patients pursuing genetic testing (Table 1). More patients have undergone genetic testing and risk assessment with the current GWA/BHA versions than in the history of the division of genetics over multiple decades (Supplementary Figure S1).

**TABLE 1:** Demographics by version of the GWA and BHA.

	GWA 1.0	GWA 2.0	GWA 3.0	GWA 4.0	BHA
Total patients (N)	52,902	31,091	87,575	153,995	33,609
Race, n (%)					
American–Indian or Alaska native	140 (0.3)	92 (0.3)	193 (0.2)	393 (0.3)	63 (0.2)
Asian	4,311 (8.1)	2,636 (8.5)	6,837 (7.8)	14,888 (9.7)	2,651 (7.9)
Black or African–American	2,458 (4.6)	1,842 (5.9)	3,614 (4.1)	6,602 (4.3)	1,707 (5.1)
Pacific Islander or Hawaiian native	0 (0)	46 (0.1)	94 (0.1)	173 (0.1)	30 (0.1)
Other or unknown	10,623 (20.1)	6,950 (22.4)	16,168 (18.5)	29,065 (18.9)	5,239 (15.6)
White	35,315 (66.8)	19,525 (62.8)	60,669 (69.3)	102,874 (66.8)	23,919 (71.2)
Ethnicity, n (%)					
Hispanic/Latino	3,344 (6.3)	2,772 (8.9)	5,480 (6.3)	11,803 (7.7)	1,772 (5.3)
Non-Hispanic	49,127 (92.9)	28,037 (90.2)	81,316 (92.9)	14,0376 (91.2)	31,640 (94.1)
Unknown	431 (0.8)	282 (0.9)	779 (0.9)	1,816 (1.2)	197 (0.6)
Age, n (%)					
18–39	15,114 (28.6)	9,804 (31.5)	22,464 (25.7)	44,713 (29)	268 (0.8)
40–49	10,778 (20.4)	6,296 (20.3)	15,440 (17.6)	28,287 (18.4)	7,990 (23.8)
50–64	20,386 (38.5)	11,767 (37.8)	26,681 (30.5)	43,699 (28.4)	13,773 (41)
65+	6,624 (12.5)	3,224 (10.4)	22,990 (26.3)	37,296 (24.2)	11,578 (34.4)
Sex, n (%)					
Male	20,506 (38.8)	10,389 (33.4)	32,562 (37.2)	58,930 (38.3)	4 (0)
Female	32,369 (61.2)	20,702 (66.6)	55,013 (62.8)	95,060 (61.7)	33,605 (100)

### Education and accelerating the diffusion of knowledge

Accelerating the diffusion of knowledge regarding the incorporation of genomics into medical care is critical for the success of the program. Educational videos were created for internal and external audiences covering key topics ranging from GINA and pharmacogenomics to downstream implications on screening and management. Virtual town halls were created for Q&A along with more traditional educational outreaches, such as grand round lectures and “lunch and learn” sessions for primary care offices.

Establishing the Genomic Ambassadors program enabled PMED to innovate quickly and disseminate knowledge. Approximately 10 primary care providers per year are supported by PMED to assist with generating insights, developing improvement strategies, implementing strategies, and then, reviewing data to determine success. This targeted model of a learning health system has led to ongoing incremental improvements to the program. It further amplifies the “voice” of our genomics content experts in our systems as the primary care ambassadors teach and receive input from their peers as part of the program.

### Expansion of population screening to diverse communities

The NorthShore merger with Swedish Covenant Hospital in 2020 expanded the patient catchment area to northern Chicago and provided an opportunity to expand access to an ethnically and socioeconomically diverse patient population. Since the expansion, thousands of Swedish Hospital patients have completed the GWA, and the PMED team has engaged in community-based participatory research with more diverse communities (Lemke et al., 2022).

## Results

### Mixed-methods research to better understand the patient and provider experiences

To lay the foundation for wider implementation of a PGx program, PMED investigators employed multiple methods to query PCP readiness and the patient experience. Three main themes emerged: perceived value and utility of PGx testing, challenges to implementation in practice, and provider and patient needs. PCPs expressed perceived benefits of PGx testing, such as avoiding adverse drug effects, more efficient dose titration, improved shared decision making, and the ability to provide patients with reassurance. Concerns were expressed about the privacy, cost, insurance coverage and level of knowledge regarding PGx results (Lemke et al., 2017). Patients also expressed no difference in the personal utility of PGx testing offered through a designated PGx clinic or with direct in-home testing. However, some did express privacy concerns, and most were unfamiliar with privacy protections provided by the GINA Act (Lemke et al., 2018).

Four main themes emerged regarding GWA implementation: benefits to clinical care, challenges in practice, CDS-specific issues, and physician-recommended improvements. Sub-themes emerged, such as perceived value in increased access to genetic services, time limitations to discuss GWA recommendations, lack of patient adherence with recommendations, and provider alert fatigue. These findings suggested that while PCPs valued the clinical utility of the GWA, there remained several challenges identified with its administration and use in practice (Lemke et al., 2020a).

A mixed-methods approach assessed PCP readiness and the patient experience with DNA10K. Like their PGx and GWA experiences, PCPs expressed concerns about and limited confidence with tasks related to test ordering, interpretation, and management of the results. Respondents perceived a high level of clinical utility for patients and their families, though there were logistical challenges to incorporating testing into their busy practices. PCPs were also unfamiliar with the privacy protections of the GINA and were concerned about patient data privacy and the potential for insurance discrimination. Adaptive refinements of several processes were implemented that improved the PCP experience with DNA10K (Lemke et al., 2020b). To assess the patient’s experience with the deployment of DNA10K, patients were offered an online survey 3 weeks after result disclosure and in six months. The patient reported understanding of results was high for cancer and cardiovascular disease risk variants. The overwhelming majority of patients perceived its personal utility as “high,” most patients shared the results with their families, and most patients expressed high levels of satisfaction with the process. Moreover, result-related health behaviors and discussions with PCPs increased over time, particularly for patients with “positive” test results (Lemke et al., 2021).

### Genetic screening and testing outcomes for GWA and DNA10K

As of 1 August 2023, a version of GWA (1.0-4.0) or BHA has been completed at least once by 228,766 patients (Table 1), with 35,432 patients completing genetic testing within NorthShore after having completed either the GHA or BHA. A total of 4,662 pathogenic (P) or likely pathogenic (LP) variants have been assigned to 4,084 patients with 463 P/LP variants in Centers for Disease Control & Prevention (CDC) Tier 1 conditions (HBOC, Lynch syndrome, Familial Hypercholesterolemia).

### Pharmacogenomics and medical outcomes

PMED’s growing PGx program and its relationship with the NorthShore Outcomes Research Network have made possible the evaluation of medical outcomes, including hospital admissions, readmissions, and analyses of the relationship between multimorbidity, polypharmacy, social determinants of health, and gene–drug interactions. David et al. first reported that DNA10K patients (*n* = 10,104) were significantly more likely to be readmitted within 90 days of hospital discharge if they had one or more PGx interactions with CPIC medications prescribed within 30 days of admission (odds ratio (OR) = 1.42, 95% confidence interval (CI) 1.09–1.84 (*p* = 0.01)). After adjustment for comorbidities and other covariates, the odds of readmission were increased by more than 30% for patients with one or more CPIC PGx interactions (OR = 1.32, 95% CI 1.02-1.73) (*p* = 0.04) (David et al., 2021b). In a follow-up evaluation with roughly twice the sample size, led by Saulsberry and the PMED team, we replicated these findings and also showed that social determinants of health (including race, employment status, and income) were the major drivers of hospital readmission (Saulsberry et al., 2022). In fact, the odds of 90-day readmission for patients with one or more identified gene–drug interactions after adjustment for robust SDOH and other covariates was attenuated by 10% (OR = 1.31, 1.08–1.59) (*p* = 0.006). The PMED team is currently evaluating the relationship between the most widely prescribed CPIC mediations, gene–drug pairs, and condition-specific outcomes, and our team is exploring the use of natural language processing and machine learning to improve the efficiency and fidelity of data mining of the EHR to capture adverse drug events resulting from pharmacogenomic interactions.

### Implementation of the polygenic risk score in primary care for personalized cancer screening

Leveraging research and clinical infrastructures at NorthShore, such as the GHI, large numbers of PCPs, and the EHR, we assessed the feasibility of the clinical implementation of the PRS in primary care. In a pilot study (Conran et al., 2021), we identified 281 subjects through the GHI who were 40–70 years old and without a personal history of breast, prostate, or colorectal cancer. The PRS for these cancer types was calculated and shared with participants through their primary care provider. Over 20% of these subjects received at least one high PRS for these three types of cancer. Many of these subjects did not have any known family history and otherwise would not realize their increased risk.

## Discussion

### Lessons learned from a decade of adoption and implementation

Starting with a needs assessment of physician stakeholders and expanding to mixed-methods and outcomes research, NorthShore’s experience with patient-centered formative research has driven an iterative process of rapid adoption and implementation in a learning healthcare system. Iterative learning from patients, providers, and leadership and community stakeholders has fueled a virtuous cycle of activation, innovation, validation, adoption, adaptation, and renewal symbolized as “activate–input–discover–validate” (Figure 3). Sharing our experience is key to the advancement of clinical genomic medicine and is a foundation for the diffusion and implementation of future technologies.

There were challenges and critical inflection points in the evolution of PMED. Key lessons learned include ensuring there is continuous wide stakeholder engagement, periodic reassessment of the educational needs of patients and clinicians, and the importance of developing partnerships between clinical and administrative champions for PMED within the organization. Defining what personalized medicine meant at NorthShore was important in developing the roadmap. Initially, PMED focused on PGx and then evolved to germline risk assessment, which was complementary to pathology efforts in oncology. To ensure success, an organization needs to have administrative and clinical alignment on *what* they are implementing.

Educating and building acceptance in a targeted area of implementation is critical stakeholder buy-in. Primary care education on *why* genomics should be important to the busy primary care clinician was critical for PMED. At the core, tackling the concept of “genetic exceptionalism” was critical, and debunking the notion that genetics is “too complicated” or “esoteric” compared to other areas of medicine for primary care was necessary. Over time, as CDC Tier 1 conditions, NCCN, and U.S. Preventive Services Task Force (USPSTF) guidelines continue to expand, the importance of genomics and family history screening from a primary care perspective is becoming more defined.

Finally, understanding the business value proposition is paramount for long-term success. The balance of “fee for service” to “value-based” care will differ depending on the organization and, thus, change the inputs and outputs for a financial model for an organization contemplating a personalized medicine program. This can impact the decision on whether to implement first in primary care or within certain specialties, such as cardiology and oncology. The key deliverable is to bring the appropriate financial stakeholders to the discussion when exploring plans for a personalized medicine program.

Standing at the forefront of personalized medicine implementation, we seek to square up to a range of remaining challenges and implementation gaps. At each step of the GWA process, from completing the tool to follow-through on recommended screening and prevention, there is attrition. The balance between having an “easy button” solution *versus* one that requires more engagement of the PCP is critical as primary care endorsement was the most impactful driver to have a patient move to the next step. Keeping patients engaged and informed of their genetics over time poses additional challenges. While future testing may be required as technology evolves, the data currently available can provide insight over a lifetime and evolve with new medical knowledge. For example, the *NBN* c.657_661del (p.Lys219fs) variant has had changing NCCN recommendations regarding its impact on breast cancer risk (NCCN Genetic/Familial High-Risk Assessment: Breast, Ovarian, and Pancreatic Version 2.2024) (Daly et al., 2021). Dissemination of this knowledge is now possible due to the infrastructure in place but remains challenging until more robust care pathways can be deployed at different touchpoints in the healthcare system.

We join with other learning health systems across the US to share successful personalized medicine adaptations that advance health equity and are transportable to other communities.

## Data Availability

The raw data supporting the conclusion of this article will be made available by the authors, without undue reservation.
