# Supplementary material for: Personalized medicine in a community health system: the NorthShore experience
Source: Front Genet. 2023 Nov 28;14:1308738. doi: 10.3389/fgene.2023.1308738 (PMC10713750; doi:10.3389/fgene.2023.1308738)
Supplement: Supplementary file 1 [file Image1.pdf]

# *Supplementary Material*

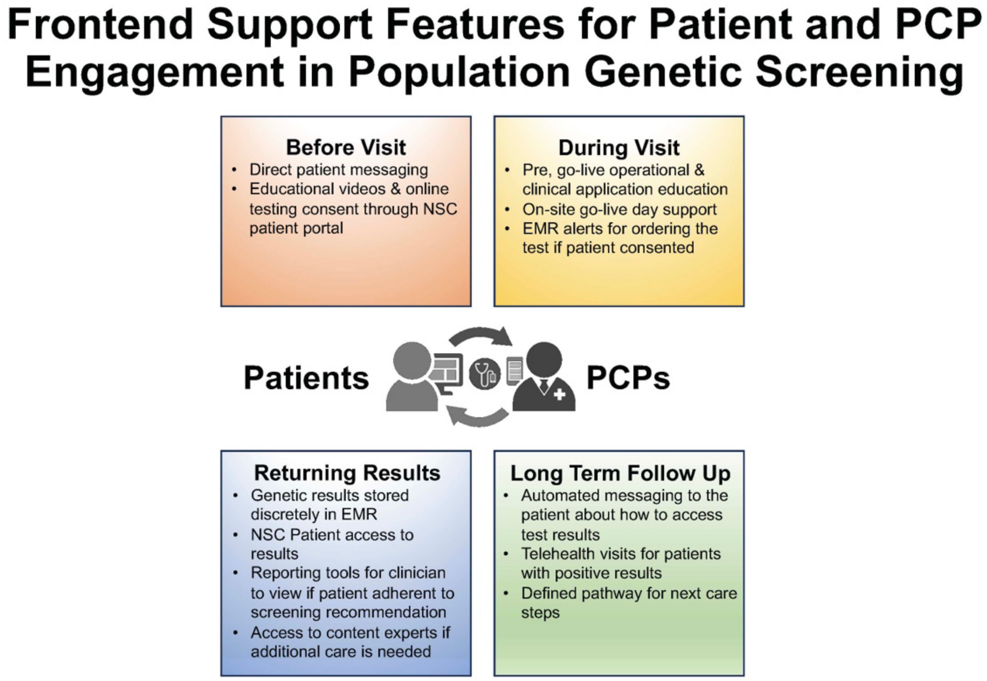


**Supplemental Figure 1: Frontend Support Features for Patient and PCP Engagement in Population Genetic Screening**

**Legend:** The NorthShore Center for Personalized Medicine program implemented multiple components to support widespread PCP adoption and engagement. (a) patient educational video and consent via the NorthShore Connect (NSC) patient portal; (b) PCP electronic clinical decision support and EHR ordering; (c) access to a NorthShore lab for phlebotomy; (d) In-house or outsourced (e.g., Color Genomics, GeneDx) NGS and PGX testing panel; (e) discrete reporting of results available in the EHR; (f) coordination of results with PCP health maintenance visits; (g) automated patient messaging about next steps; (h) access to genetic counseling; (i) return of results on the patient portal; and (j) clinical follow-up with PCP and specialists. Abbreviations: Primary care physician (PCP); electronic medical record (EHR)

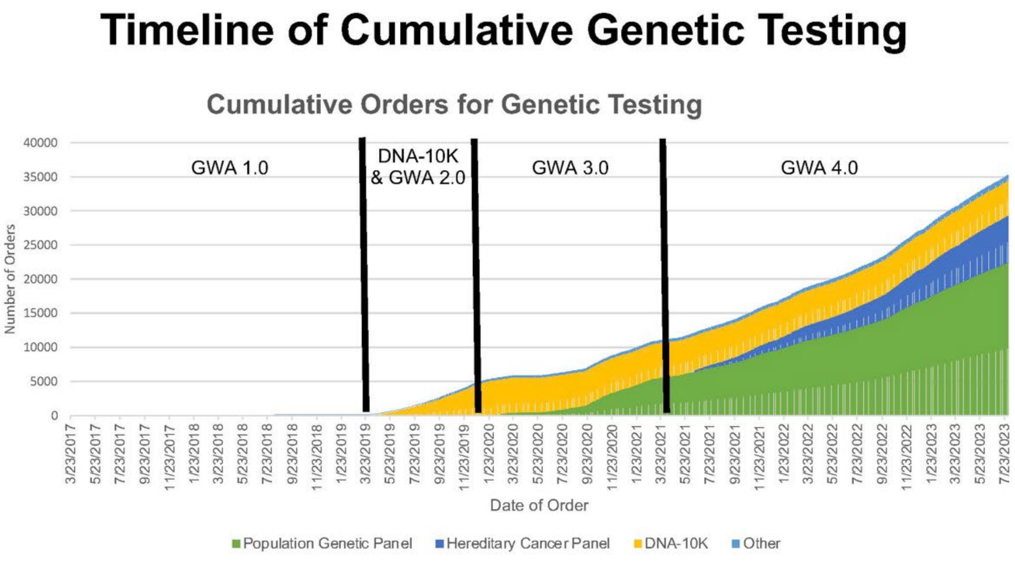


**Supplemental Figure 2: Timeline of Cumulative Genetic Testing**

**Legend:** Growth of genetic testing across the spectrum of PMED primary care initiatives involving iterative versions of GWA, BHA, and DNA10K.
